# Bridging the Gap: Including Patient Voices in Short-Term Medical Mission Evaluations

**DOI:** 10.5334/aogh.2431

**Published:** 2019-06-18

**Authors:** Diana Morales, Wendy Clay, Rebecca Khamishon, Rachel Zaragoza, Reem Eldnaf, Alison Trautman Nagy, Michael Ong, Jiro Morales, Mark Ryan

**Affiliations:** 1 VCU School of Medicine 2021, US; 2 VCU School of Medicine 2022, US; 3 VCU School of Pharmacy 2021, US; 4 VCU School of Pharmacy 2020, US; 5 VCU Department of Family Medicine and Population Health, US

## Abstract

**Background:**

Several studies have evaluated short term medical missions (STMM), but most have been from the perspective of the STMM teams providing their own suggestions [1]; few surveys have assessed the perceptions of patients who seek medical care at these STMM clinics [2].

**Objectives:**

This project evaluates the efficiency, quality, and value of the services provided by a STMM clinic established in the community of Paraíso in Santo Domingo Norte, Dominican Republic, as perceived by the patients. Study results will allow organizers to make improvements to these clinics and will contribute to a broader understanding of the benefit and value of medical services provided by STMMs.

**Methods:**

A mixed method, cross-sectional survey was created which consisted of 11 questions and a medication quiz. The survey questions were based on items used in prior surveys, as well as the participant responses to those surveys, and attempted to address perceived efficiency, quality, value, and effectiveness of services provided, as well as the impact on the community.

**Findings:**

Two-hundred sixty-six patients were invited to complete the survey, and 117 (44%) were enrolled in the study. The majority of survey responses were positive and highlighted patients’ satisfaction with provider skills and communication. Of note, many responses identified longer than anticipated wait times during two portions of the patient encounter and self-reported deferral of local care to receive care with the STMM. Additionally, although average medication quiz scores were high, average scores decreased with age.

**Conclusions:**

This study brings to light patient perceptions of services at a STMM in the community of Paraíso in Santo Domingo Norte, Dominican Republic. Survey responses highlight the importance of efforts to: minimize clinic wait time; enhance collaboration between local providers and STMMs to reduce deferral of care; and improve medication knowledge among the geriatric population.

## Introduction

Short-term medical missions (STMMs) can be generally defined as grassroots initiatives to provide low cost or free medical care to lower and middle-income countries [1]. Studies show that the number of STMMs as well as the number of participating physicians are continually increasing [1], with the United States as one of the leading sending countries [2]. According to one estimate, the global cost expended on STMMs from the US exceeds $3.7 billion [1]. Along with this growing participation and expenditure, however, there has been growing concern regarding common criticisms of STMMs. In an extensive review of articles about STMMs between 1985 and 2009, Martiniuk et al. [2] found that many had raised concerns over limited mission impact, sustainability, cost effectiveness, quality of care, and lack of preventive measures. Sykes noted that despite increased efforts on measuring the impact of STMMs, empirical evaluation was still limited [3]. Even more troubling, most studies evaluating STMMs have examined only the perspective of providers and mission organizers, rather than the recipients of care [4]. In one study surveying health professionals, all the respondent groups cited language proficiency and cultural awareness as important for the success of STMMs, but many also recognized their own limited capabilities in these areas [5]. This shows that providers are aware of deficiencies in communication with patients, though they may not be aware of how this is impacting patient care. There is little published data on how STMMs might engage with recipients of care beyond addressing language services in order to assess patient needs and quality of care. Unless providers and STMM organizers seek input from patients, there may be issues with the services of which the STMMs are unaware, and which remain unresolved. This gap in published data adds to the importance of creating a patient centered evaluation of STMMs.

The few prior studies assessing patient perception of STMMs have revealed which elements patients value most about STMMs and how this perception affects their utilization of services. One study consisted of semi-structured interviews with patients receiving care from an STMM in the Dominican Republic [4]. Within their responses, participants identified certain factors of STMMs that affected access to care, including lower cost, greater availability of health care workers, access to medicines, and convenient location. This shows that patients believe STMMs are valuable for communities where there are few healthcare resources available. The authors also noted that participants seemed reluctant to mention any difficulties with communication, perhaps due to the presence of interpreters and STMM staff during the interview, which “suggests a need for programmes not only to ask about miscommunication but also to develop ways to monitor for it proactively [4].” A study surveying patients at a Taiwanese STMM to Swaziland had similar responses [6]. The majority of participants reported high satisfaction, and almost all hoped to see STMM services provided in the future. The study also provided insight into effective communication and use of interpreters; most responded that they understood the providers’ explanations as well as how to treat their condition, and nearly all could follow the providers’ instructions. There were some limitations to the survey, however: the authors noted that the answer choices—sometimes limited to “yes or no”—may not provide sufficient detail regarding patient perception. Finally, a study that found patients primarily chose to seek care at a surgical STMM site in Guatemala because of its reputation for high quality of care; affordability was the second most common reason [7]. Although the study did not address which elements patients deemed to be “high quality,” there was some concern that this perception may have led some patients to delay surgical treatment, believing that they would receive better care from the STMM providers than from local providers. This would suggest that STMMs had the potential to cause unintended harm, thus emphasizing the need to include recipients of care in STMM evaluations.

It is clear that there are still gaps in the research on STMMs, especially regarding patient perspectives. Previous evaluation of STMMs have laid a foundation upon which others can now build and improve. Humanitarian Outreach Medical Brigade Relief Effort (HOMBRE) is a 501c3 status, non-profit organization that has travelled to the Dominican Republic since 2009 in partnership with another 501c3 non-profit, the Dominican Aid Society of Virginia (DASV), to provide primary care services on STMMs [8]. The STMM consists of a Triage station (patient registration and documentation of their chief complaint and vital signs), a Consultorio station (where the clinical interview, exam, and assessment occur), and a Pharmacy station (where patients receive medications and information on self-management for their diagnoses). The community of Paraíso (in Villa Mella, just north of the capital of Santo Domingo) receives care twice a year from groups of volunteer providers. The aim of this study was to evaluate patient perceptions of the efficiency, quality and value of services provided by STMM clinics in the community of Paraíso in Santo Domingo Norte, Dominican Republic, and to begin evaluating the quality of STMM team-patient communication.

## Methods

### Study Participants

A cross-sectional survey was conducted among adult patients of an STMM clinic located in Paraíso, Santo Domingo Norte, Dominican Republic, June 6–10, 2018. Eligible adults between the ages of 18 and 89 years were invited to complete the survey following their medical care at Escuela Comunal Altos de Paraíso. Eligible adults who opted out of the survey were excluded from the study, as were children under 18 and adults over 89 due to privacy requirements. This study was approved by the Virginia Commonwealth University Institutional Review Board (IRB) in Richmond, Virginia (HM20013064). Per communication with the Dominican Republic’s Consejo Nacional de Bioética (CONABIO), no review was required by this organization given that this project is non-interventional/epidemiological in nature.

### Methods

**Figure 1:**
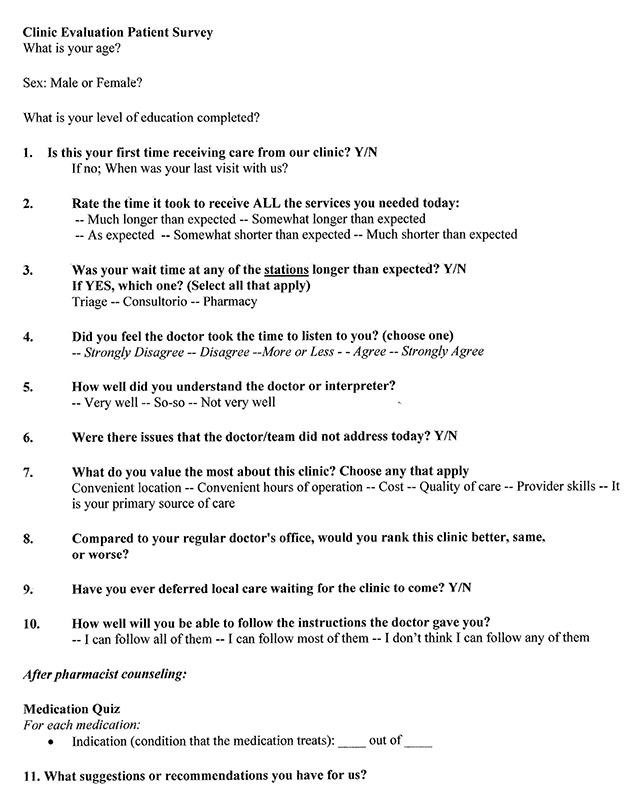
Cross-sectional Survey Administered in the Dominican Republic.

Community members waiting to be seen by the clinic were informed of the survey through a public announcement made at the beginning of clinic. The survey was administered at the completion of patients’ clinic visits, following the pharmacy consult. If a subject expressed interest in participating, a standardized verbal consent was read. Age was confirmed to ensure the participant was eligible, and the survey was administered verbally. A medication quiz was given as part of the survey, in which participants were asked to review the medications they received with pharmacy counseling, and to identify the indication for each medication. This quiz was scored as a percentage of correctly identified medications, and a teach-back method was used if the score was less than 100%. A copy of the survey can be seen in Figure 1. All surveys were administered either by Spanish-proficient STMM team members, or by English-speaking team members with Dominican medical students and graduates serving as volunteer medical interpreters.

### Study Design

A mixed method (quantitative and qualitative) cross-sectional survey was created, which included 11 questions and a medication quiz. The qualitative question “what suggestions or recommendations do you have for us?” was used as personal feedback for the clinic and was not analyzed for research purposes. Questions were based on items used in prior surveys, as well as the participant responses to these surveys [4,7,9]. Measures included age, gender, level of education, and questions asking patients to rate various aspects of clinic care and logistics (Figure 1). All questions were yes/no, Likert scale, or multiple-choice format. Questions were designed for subjective assessment of the clinic quality of care. A medication quiz was designed as an objective assessment of the quality of care provided by the STMM.

### Statistical Analyses

Data was compiled with percentages and means calculated using Microsoft Excel. Bivariate analyses were conducted using cross tabulation with Chi-square test comparing dichotomous/categorical variables. The test was conducted with SPSS (©IBM Inc. Version 25, 2017). Cross tabulation with Chi-square was used to examine associations between clinic satisfaction and being a first-time patient at the clinic as well as education and age with level of understanding the doctor. Participants were stratified into four age groups (18–24, 25–44, 45–64, 65+).

## Results

Two-hundred sixty-six patients were invited to complete the survey, and 117 (44%) were enrolled in the study. Of the study participants, 37 were male and 80 were female. Ninety-five participants were over the age of 45, and 95 were returning patients. Additionally, 81 participants had an 8th grade education or lower (Table 1).

**Table 1:** Patient Demographics.*

	Number (%)		

	Male	Female	Total

	37 (32%)	80 (68%)	117 (100%)

Age			
18–24	0 (0%)	4 (3%)	4 (3%)
25–44	3 (2%)	15 (13%)	18 (15%)
45–64	16 (14%)	31 (26%)	47 (40%)
65+	18 (15%)	30 (26%)	48 (41%)
Patient Status			
New Patient	2 (2%)	20 (17%)	22 (19%)
Returning Patient	35 (30%)	60 (51%)	95 (81%)
Education Level			
None	6 (5%)	12 (10%)	18 (15%)
Primary (1–6)	16 (14%)	30 (26%)	46 (40%)
Intermediate (7–8)	6 (5%)	11 (9%)	17 (14%)
High (9–12)	6 (5%)	16 (14%)	22 (19%)
Some College	2 (2%)	7 (6%)	9 (8%)
College Degree	0 (0%)	2 (2%)	2 (2%)
Graduate Degree	1 (1%)	2 (2%)	3 (3%)

* Percentages represented out of N = 117.

**Figure 2:**
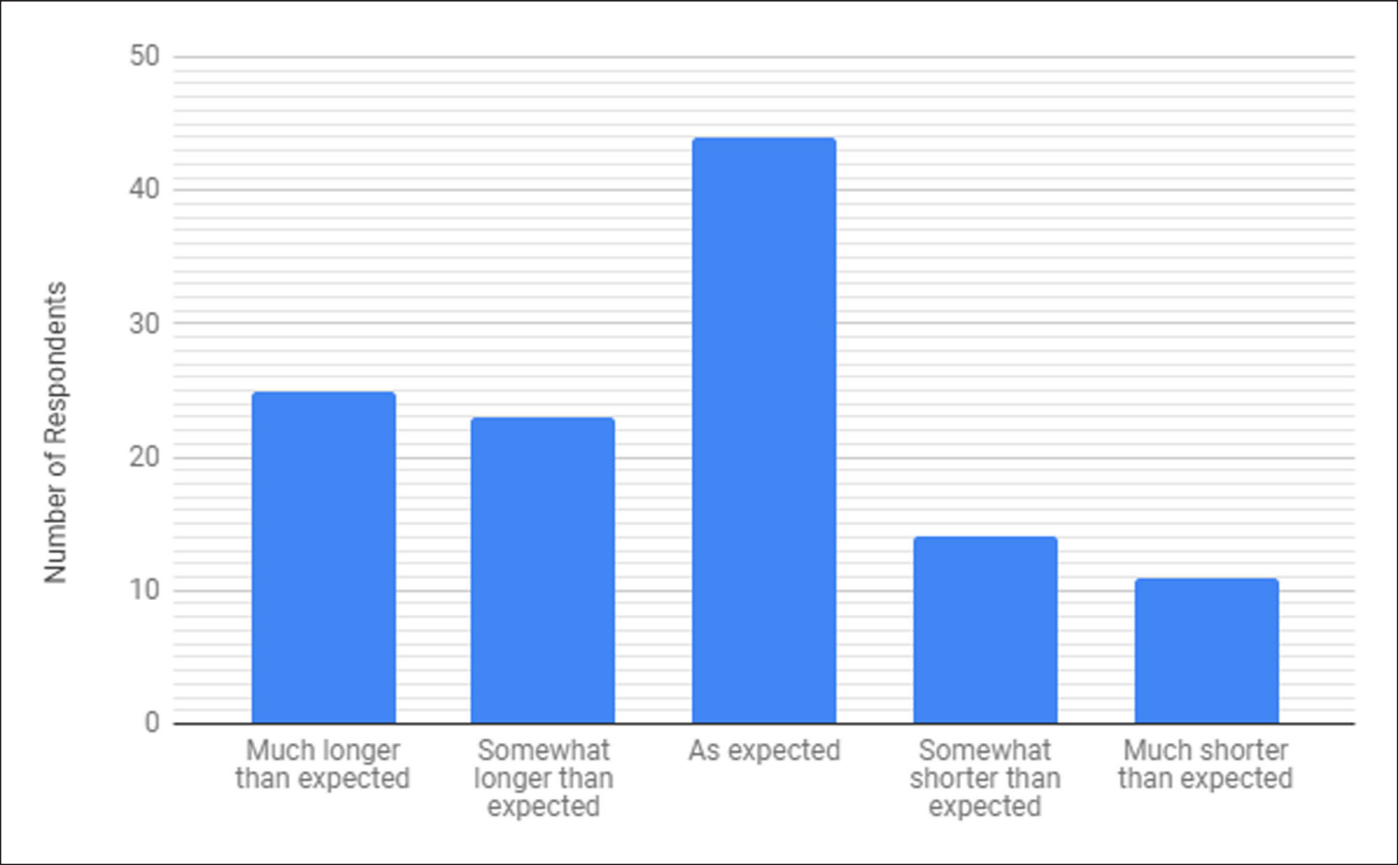
Perceived Efficiency of Clinic Wait Time.

Survey questions fell within one of five broad categories: perceived efficiency of services provided, perceived quality of services provided, perceived value of the services provided, impact on the community, and effectiveness of the services provided. With regards to efficiency, 41% of all respondents felt that their wait time was longer than expected to some degree while 59% of individuals felt that wait time was as expected or shorter than expected (Figure 2). Forty percent of returning patients and 45% of new patients felt that the wait time was longer than expected. Thirty-six percent of respondents felt that a specific station within the clinic had a longer wait time than expected and of these, 52% indicated Consultorio and 45% selected Triage as the stations with longer wait times than expected (Table 2).

**Table 2:** Perceived Efficiency of Clinic Wait Time.*

Wait time perceptions	Frequency (%)		

	Longer	As Expected	Shorter

Age			
18–24	4 (3%)	0 (0%)	0 (0%)
25–44	10 (9%)	5 (4%)	3 (3%)
45–64	16 (14%)	20 (17%)	11 (9%)
65+	18 (15%)	19 (16%)	11 (9%)
Total	48 (41%)	44 (38%)	25 (21%)
Patient status			
New patient	10 (9%)	4 (3%)	8 (7%)
Returning patient	38 (32%)	40 (34%)	17 (15%)

Longer than expected station wait time?	**Yes**	**No**	
	42 (36%)	75 (64%)	

Which station took longer?	**Triage**	**Consultorio**	**Pharmacy**
	19 (16%)	22 (19%)	3 (3%)

* Percentages represented out of N = 117.

In terms of quality, the major focus was on provider communication. Ninety-seven percent of respondents felt that the physicians took the time to listen to them and 93%, regardless of education level, felt that that they understood their doctor/interpreter very well (Table 3). Only 8% of respondents felt that their physician did not address an issue they had wanted addressed (Table 3).

**Table 3:** Survey Responses Regarding Provider Communication.*

	Frequency (%)				

Did your physician take the time to listen to you?	**Strongly Disagree**	**Disagree**	**Disagree More or Less**	**Agree**	**Strongly Agree**
	1 (1%)	1 (1%)	2 (2%)	41 (35%)	72 (62%)

How well did you understand your doctor/interpreter?	**Very well**	**So so**	**Not very well**		
No Education	17 (15%)	1 (1%)	0 (0%)		
Primary	43 (38%)	3 (3%)	0 (0%)		
Intermediate	15 (13%)	2 (2%)	0 (0%)		
High School	20 (17%)	2 (2%)	0 (0%)		
Some College	9 (8%)	0 (0%)	0 (0%)		
College Degree	2 (2%)	0 (0%)	0 (0%)		
Graduate Degree	3 (3%)	0 (0%)	0 (0%)		
Total	109 (93%)	8 (7%)	0 (0%)		

Were there any issues that the doctor/team did not address today	**Yes**	**No**			
	9 (8%)	108 (92%)			

* Percentages represented out of N = 117.

**Figure 3:**
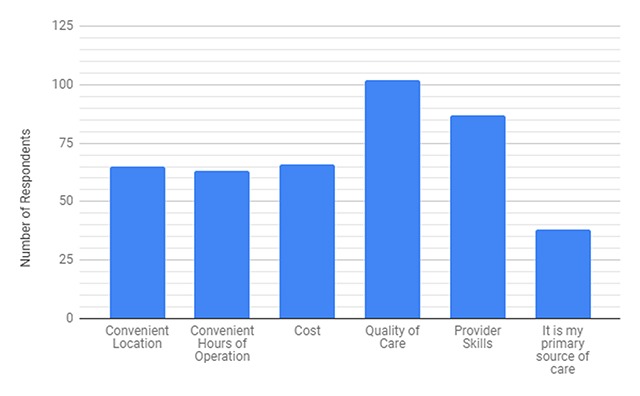
Responses to survey question “What do you value most about this clinic?”* * Numbers add to more than 117 because respondents were able to choose more than one option.

When looking at what each respondent valued most about the clinic, factors related to quality dominated, with 87% of respondents answering quality of care and 74% of respondents answering that provider skills was valued most (Figure 3). Additionally, 70% ranked the clinic as better than their regular doctor’s office (Table 4). Community impact was assessed through the number of patients who deferred care waiting for our team to come back, and 48% of respondents indicated that they had deferred care at some point (Table 4).

**Table 4:** Survey Responses Regarding Quality and Community Impact.*

	Frequency (%)		

How does this clinic compare to your regular doctor’s office?	**Better**	**Same**	**Worse**
	82 (70%)	34 (29%)	1 (1%)
Have you deferred local care waiting for this clinic?	**Yes**	**No**	
	57 (49%)	60 (51%)	

* Percentages represented out of N = 117.

**Figure 4:**
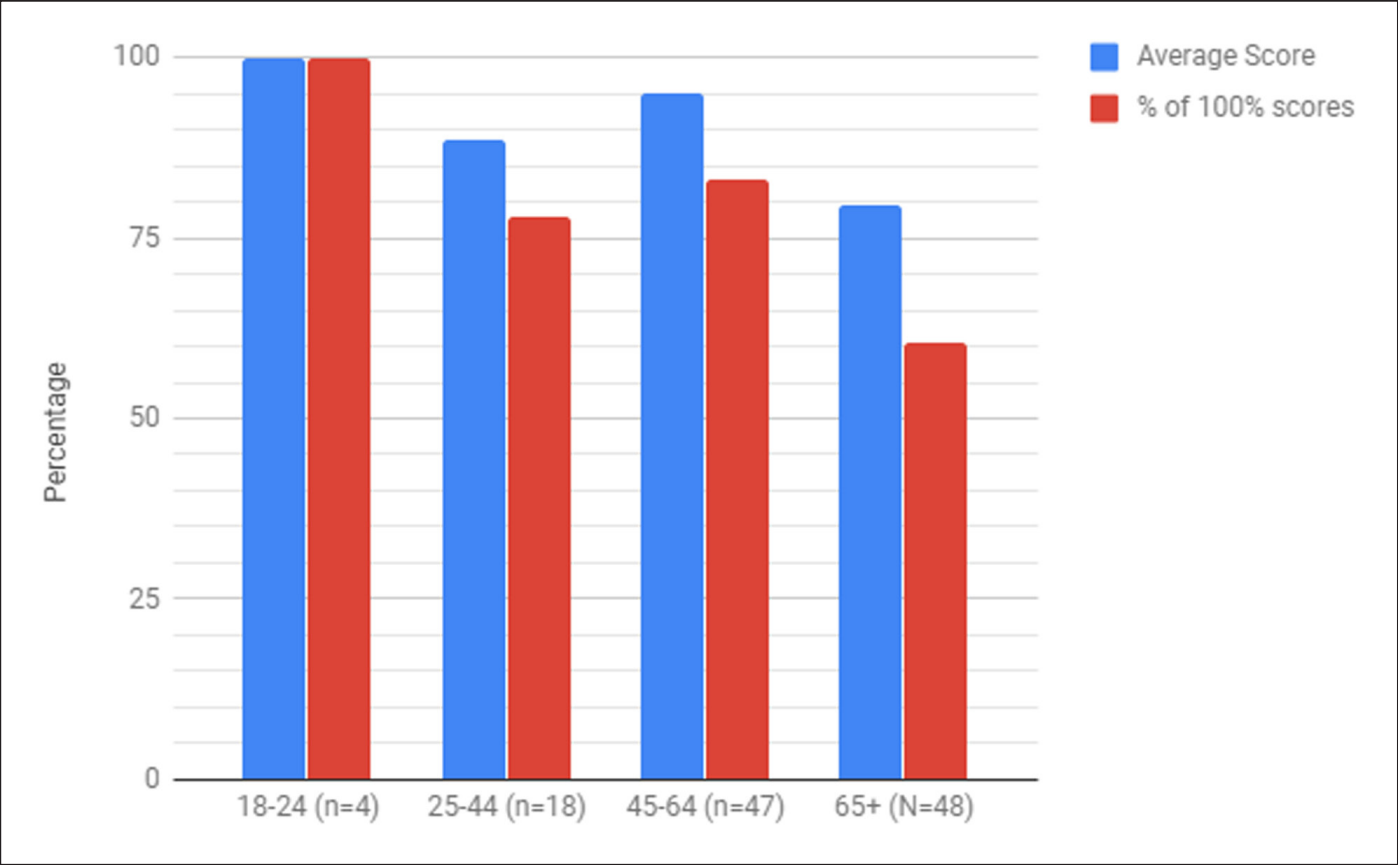
Representation of Medication Quiz Scores within Age Groups.

The final survey questions sought to examine how effectively our clinic met the health needs of respondents by gauging how well they believed they would be able to follow the instructions given to them, as well as how well they understood the information given to them about their medications. Ninety-five percent of respondents felt that they would be able to follow all of the instructions given to them, and the average medication quiz score was 91% (Table 5). Average quiz scores decreased with increasing age (Figure 4). Of note, 73% of respondents scored 100% on the quiz, but of these, 66% were under age 65. Additionally, new patients had lower quiz averages (82%) when compared to return patients (92%) (Table 5).

**Table 5:** Survey Responses Regarding Meeting Patient Needs: Following Instructions and Medication Quiz Score (stratified by age, education, and patient status).*

	Frequency (%)						

**How many of the instructions given to you can you follow?**	**All**	**Most**	**None**				
	88 (75%)	28 (24%)	1 (1%)				

**Average medication quiz score**							
	**18–24**	**24–44**	**45–64**	**65+**	**All**		
Average score based on age	100%	88.42%	95.08%	79.48%	91%		
# of respondents in each age group scoring 100%	4 (3%)	14 (12%)	39 (33%)	29 (25%)	86 (74%)		
Average score based on education level	**No Education**	**Primary**	**Intermediate**	**High School**	**Some College**	**College Degree**	**Graduate Degree**
	90%	90%	86%	92%	98%	100%	100%
Average Score Based on Patient Status	**New Patient**	**Returning Patient**					
	82.28%	92.82%					

* Percentages represented out of N = 117.

## Discussion

The purpose of this study was to fill the gap in research on STMMs and create an evaluation to achieve a better understanding of patients’ perspectives of care received. Our results indicate there are several areas in which the clinic excels and other areas where there is room for improvement.

The data suggests the clinic excels at providing patients with high quality care. Patients reported that once in the medical visit with the provider, they were highly satisfied with the communication and the provider engagement in their care. This suggests that patients had their main concerns appropriately addressed during their clinic visits. Patients also identified the quality of care provided as their main reason for utilizing STMM clinic services, and also identified convenience, cost, and provider skills as important considerations in attending the STMM. Interestingly, nearly two-thirds of patients felt that the STMM experience was favorable to the experience in their usual site of care (Table 4), but the survey was unable to clarify why this was the case.

Objective assessment of the quality of care was assessed by a medication quiz at the conclusion of the survey. Higher quiz scores may argue for better quality of care and patient/provider communication in that it would suggest better retention of the information given in the provider visit and via pharmacy counseling. The high average quiz scores that were obtained suggest that overall patient perceptions of high-quality care are valid, given the high level of retention of information provided during their STMM encounter.

Using the teach-back medication quiz, it is possible to identify certain patient characteristics which resulted in lower recall of medication indications. The data suggests a correlation between a lower quiz score and patients’ ages over 65 (Figure 4). A confounding factor in this result could be the greater number of medications used by the geriatric population, which would make recall more difficult. A study by Sergi et al. [10] found that in the United States polypharmacy is a growing issue in the geriatric population. Levels of polypharmacy amongst the geriatric population have been shown to vary between different low and middle income nations [11,12], but as provider adherence to recommended treatment improves and patients gain access to those treatments, prevalence has been increasing [12]. Future areas of study may find ways to improve handling care for patients 65 years old and older. Some options could be spending more time with patients, providing other cues to help clarify medication indications and uses (e.g. color-coding blood pressures, or using symbols to help identify medication uses during pharmacy counseling). Future research should highlight the implementation of a patient education plan in order to help this vulnerable population.

In addition, the medication quiz indicated that first time patients to the clinic had a lower average score versus returning patients (Table 5). The familiarity that past patients have with the clinic could account for this statistic. This increased recall for patients who had attended the STMM in the past may also be enhanced by the use of an electronic health record (fEMR, https://teamfemr.org/) to facilitate follow-up care, thus helping ensure that patients are prescribed medications they are already taking, and with which they may already be familiar. Also, this STMM has used a single in-country source for medications to treat chronic illnesses, and patients may have become familiar with medications names, packaging, and appearance. Finally, new patients may not have been on medications previously, making the new information more difficult to retain. Future STMMs should establish a method to identify new patients or patients with multiple medications, in order to provide detailed explanations to help them understand treatments and potentially improve adherence.

As previously stated, patient satisfaction data suggests that patients feel that this STMM provides high quality care. However, there are areas for improvement, including the wait time to receive clinical services. Waiting to be seen by the provider in the clinic room (the Consultorio) was identified as a principal site of delay (Table 2). This is likely the result of the limited number of volunteer providers, combined with a desire to provide as comprehensive a level of care as possible, and a goal of seeing as many patients as could be accommodated during each STMM clinic day. Aside from increasing numbers of volunteer providers or further limiting the number of patients seen each day, patients’ concerns about wait time might be managed by more accurately setting expectations at the beginning of the day, especially among younger patients who expressed very high levels of dissatisfaction with their wait times. One example might be to register patients for morning or afternoon sessions, thereby allowing those with afternoon slots to leave the clinic and address other needs without worrying that they will lose their place. Another option might be to consider how to prioritize patients with the greatest needs, or those who might have more difficulty tolerating longer waits (e.g. patients 65 years or older, patients 2 years or younger, pregnant women, working adults, etc.). It is important, then, to discuss what STMMs can do to set reasonable patient expectations in a setting with limited resources and high patient volume to ensure patients are not dissuaded from receiving needed screenings and services.

Since STMMs are a considerable economic investment [1], it is important to evaluate whether these trips may have any unintended consequences on underserved populations. Our data shows that many patients rank the clinic better than their regular doctor’s office and that one-third of patients state that the clinic is their primary source of care (Figure 3). Interestingly, nearly one-half of patients reported that they have deferred care available in local settings while waiting for the STMM team to arrive (Table 4). This raises a concern that patients may not utilize their local healthcare resources, and may have needs which go undetected or inadequately treated. Esquivel et al. [7] lends support to this point as it was suggested that patients were delaying surgery at local hospitals in order to receive care from an STMM. This study of the surgical STMM in collaboration with Hospital de la Familia in Guatemala suggested skewed patient perception that STMMs provide superior quality of care compared to the local health system. In another survey study by DeCamp et al. [4] the results indicated that patients strongly associated STMM with foreign aid rather than identification with a specific organization. This finding raises an ethical point in the role of STMMs in developing nations where the patients’ perception that STMMs offer better care than local resources may not be grounded in fact, or may lead to distrust or underuse of the local health system. Further investigation of this point should more directly assess any reasons for why patients may defer local care, in order to avoid STMM services having the contradictory effect of reducing patients’ attending to health needs. STMMs may play a role in helping enhance connections between patients and local providers of care to encourage management of chronic illness.

The primary limitation is the fact that there was no randomization of patients who were invited to participate in the survey, and it is possible that patients who were willing to take the time to complete the survey were those who felt they had a more positive experience. If this were the case, patient satisfaction and patient understanding of the information provided during the STMM encounters may be overstated. As a result, a significant limitation to this research, and a challenge for STMMs overall, may be the risk of response bias. Although survey administrators were not included in the clinic flow as part of the data collection, it would have been clear to the participants that these survey administrators were part of the STMM. As prior studies have noted that participants may assume that survey administrators are part of clinic staff [4], and therefore prompted to give more positive evaluations, the survey and study design were unable to assess whether the strategy of separating survey administrators from clinical stations was effective in impacting potential bias. Also, the study was not designed to assess patient satisfaction based on whether patients’ expectations for certain medications or treatments were met. Each of these factors risk overstating the positive impression patients may have of the care provided by the STMM, as unsatisfied or unhappy patients may have declined to participate. Although this study represents one of the few self-assessments of the quality of care provided by STMMs in developing nations—an assessment gap which STMMs should address—it is clear that future efforts may need to focus on further ensuring that patients feel empowered to answer survey questions without worry of negative consequences.

The research conducted did have additional limitations. The patient population surveyed was limited to the clinic in one barrio near Santo Domingo, which may limit the generalizability of our findings. Notably, the Creole population who attended the clinic spoke limited Spanish and were unable to participate in the survey. Additionally, the survey question regarding deferred medical care was difficult for patients to understand despite clarification by the research team, and this data should be considered with caution. This would be an important topic for additional research.

## Conclusion

The aim of the study was to examine patient perception of the quality and effectiveness of a twice-yearly STMM in the community of Paraíso in Santo Domingo Norte, Dominican Republic. This research further adds to the ongoing investigations of STMMs and their impact on the communities they serve. Our data shows that the clinic excels at providing a high quality of care from the patients’ perspective. Furthermore, patients are especially satisfied with the care they receive from the STMM providers, and objective findings from the medication quiz score further support this point. The data suggests that longer and more dedicated periods of time should be spent with new patients and geriatric populations to ensure understanding of their conditions and corresponding medications. However, patient responses also revealed problems with the flow of the clinic, and important concerns that the clinic could have unintended negative consequences, such as deferring medical care.

As one of the few studies examining the patient’s point of view, this research provides a basis for integrating patient input into the evaluation of STMMs in order to assess the impact of the communities they serve. Moreover, it focuses on addressing chronic illness through sustainable, ongoing relationships, compared to surgical mission trips which focus on discrete interventions with focused follow-up. Overall, this study adds to the growing body of research on STMMs and its implications on the future of global medicine.
